# Somatic mosaicism of CIAS1/NLRP3 gene in two patients with chronic infantile neurologic, cutaneous, articular syndrome

**DOI:** 10.1186/1546-0096-12-S1-P84

**Published:** 2014-09-17

**Authors:** Serena Ifmach-i Pastore, Alberto Tommasini, Silvia Borghini, Marco Gattorno, Loredana Lepore

**Affiliations:** 1 University of Trieste, Trieste, Italy; 2 Institute for Maternal and Child Health - IRCCS Burlo Garofolo –, Trieste; 3 Genetic Laboratory, “G. Gaslini” Institute for Children and Department of Pediatrics, Genoa, Italy; 4 II Division of Pediatrics, “G. Gaslini” Institute for Children and Department of Pediatrics, Genoa, Italy

## Introduction

CINCA syndrome (chronic, infantile, neurological , cutaneous, articular syndrome) also known as neonatal-onset multisystem inflammatory disease (NOMID) represents the most severe form of cryopyrin-associated periodic syndrome ( CAPS). This condition was found to be associated with missense mutations in the CIAS1/NALP3/PYPAF gene, which encodes cryopyrin. Cryopyrin is a member of the cytoplasmatic protein family CATERPILLER, which is involved in inflammasome molecular platform assembly, leading to inflammatory immune response and apoptosis regulation. It has been hypothesized that mutant cryopirin spontaneously oligomerizes and induces the inflammasome activation with elevated IL-1β production and autoinflammatory phenotype. Causative CIAS1 mutations have been detected in only half of the patients with CINCA and the genetic cause of the disease in patients who tested negative remains unclear.

## Objectives

We describe two patients with severe CINCA syndrome who exhibited somatic CIAS1 mutations , with different percentage of mosaicism.

## Methods

Case 1: female with urticarial skin rash since the first days of life, fever spikes since age of six months responsive only to steroids, knee arthritis from age 2 until age 4 years. During the follow up she developed papillema, persistent headache with mild cerebral atrophy at magnetic resonance, patella overgrowth and at age of 8 years perceptive deafness. Laboratory data always showed increased acute phase reactants. She was dependent on glucocorticoids to control her fever but no other symptoms until the age of 17, when she could be treated with anakinra and afterwards canakinumab with good clinical and laboratory response. Only perceptive deafness remains stable in spite of anti-IL1 therapy.

Case 2: female with widespread urticarial rash at birth who progressively developed spleen, liver, lymphonodes enlargement, recurrent coxalgia, a large lytic proximal tibial lesion histologically diagnosed as benign chondroid dysostosis; at age of 7 years a mild intellectual damage emerged; cerebral magnetic resonance was normal. Laboratory data were always abnormal with increased acute phase reactants. She reached complete remission after anti IL1 therapy (anakinra, followed by canakinumab).

## Results

Conventional mutation analysis of all exons of CIAS1 filed to evidence any mutation in the two patients. A new mutation analysis was performed by dr. Arostegui (department of immunology, Hospital Clinic, Barcelona, Spain) to detect the allelic frequency of possible somatic mutations in exon 3 of CIAS1. The 1688 A>G mutation was found with a 6.5% allelic frequency in patient 1 and the c1298 C>T mutation was found with a 3.2% allelic frequency in patient 2.

## Conclusion

In conclusion, we described two patients with typical CINCA syndrome due to somatic mosaicism of CIAS1. Such somatic mosaicism could be easily missed with the traditional sequencing of CIAS1. Notably, gain of function cryopirin mutations are able to provoke a strong inflammatory phenotype even when presents in mosaicism only in a small percentage of immune cells.

## Disclosure of interest

None declared.

